# Toll-like receptors and nuclear factor kappa B signaling pathway involvement in hepatorenal oxidative damage induced by some food preservatives in rats

**DOI:** 10.1038/s41598-023-32887-9

**Published:** 2023-04-12

**Authors:** Yasmina M. Abd-Elhakim, Amany Behairy, Mohamed M. M. Hashem, Khaled Abo-EL-Sooud, Abeer E. El-Metwally, Bayan A. Hassan, Haytham A. Ali

**Affiliations:** 1 Department of Forensic Medicine and Toxicology, Faculty of Veterinary Medicine, Zagazig University, Zagazig, 44519 Egypt; 2 Department of Physiology, Faculty of Veterinary Medicine, Zagazig University, Zagazig, 44519 Egypt; 3 Department of Pharmacology, Faculty of Veterinary Medicine, Cairo University, Giza, 12613 Egypt; 4 Pathology Department, Animal Reproduction Research Institute, Giza, 3514805 Egypt; 5 Pharmacology Department, Faculty of Pharmacy, Future University, Cairo, 11835 Egypt; 6 Department of Biochemistry, Faculty of Veterinary Medicine, Zagazig University, Zagazig, 44519 Egypt; 7 Department of Biochemistry, Faculty of Science, University of Jeddah, Jeddah, 23218 Saudi Arabia

**Keywords:** Biochemistry, Biomarkers, Diseases, Nephrology

## Abstract

Chemical food preservatives are extensively found in various processed food products in the human environment. Hence, this study aimed to investigate the effect of long-term exposure to five food preservatives (potassium sorbate (PS), butylated hydroxyanisole (BHA), sodium benzoate (SB), calcium propionate (CP), and boric acid (BA)) on the liver and kidney in rats and the probable underlying mechanisms. For 90 days, sixty male albino rats were orally given either water (control), 0.09 mg/kg b.wt BHA, 4.5 mg/kg b.wt PS, 0.9 mg/kg b.wt SB, 0.16 mg/kg b.wt BA, or 0.18 mg/kg b.wt CP. Liver and kidney function tests were assessed. Hepatic and renal oxidative stress biomarkers were estimated. Histologic examination analysis of liver and kidney tissues was achieved. Toll-like receptors 2 and 4 (TLR-2 and TLR-4), tumor necrosis factor-alpha (TNF-α), and nuclear factor kappa-light-chain-enhancer of activated B cells (NF-κB) mRNA expression levels were measured. The results revealed that long-term oral dosing of the five food preservatives resulted in significant increases in alkaline phosphatase, alanine transaminase, aspartate transaminase, urea, uric acid, and creatinine levels. There were significant reductions in hepatic and renal antioxidant enzymes, an increase in MDA concentrations, and pathological alterations in renal and hepatic tissues. The mRNA levels of TLR-4, TLR-2, NF-κB, and TNF-α were elevated in the food preservatives-exposed groups. Conclusively, the current findings revealed that long-term exposure to PS, BHA, SB, CP, and BA has a negative impact on liver and kidney function. Furthermore, these negative effects could be mediated via oxidative stress induction, inflammatory reactions, and cytokine production.

## Introduction

Food preservation has traditionally had three goals: conserving the look of the food, preserving the nutritional properties of the food, and extending the storage life of the food^1,2^. Antioxidants and antimicrobial agents are among the almost 3000 food preservatives currently on the market^3^. Potassium sorbate (PS), butylated hydroxytoluene (BHT), sodium benzoate (SB), butylated hydroxyanisole (BHA), monosodium glutamate, potassium bromate, boric acid (BA), and calcium propionate (CP) are the most often used food preservatives^4^. Currently, concerns surrounding the health implications posed by chemical preservatives have led to the need for an extensive evaluation of their impacts, especially with long-term exposure^5^.

Butylated hydroxyanisole (BHA, C_11_H_16_O_2_, E320) is a food additive and can be added to packaging materials to protect foods inside the package by volatilizing the antioxidant^6^. It is not irritating, but its low allergic sensitization potential can produce skin responses (allergic contact dermatitis). It is possibly carcinogenic to humans^7^.

Several consumer products include potassium sorbate (PS, C_6_H_7_O_2_K, E202), which inhibits yeast and mold growth and controls the growth of certain bacteria^8^. PS is present in many foods such as cheeses, pickles, sauces, fish products, and soft drinks as an antimicrobial agent^9^, and has been shown to have mutagenic and/or genotoxic effects^10^. Both SB and PS can burden the liver, cause sensitization, and disturb children's behavior^11^.

Sodium benzoate (SB, C_7_H_5_O_2_Na, E211) is a frequently used preservative in food and beverages. It is a popular soft drink preservative because it inhibits bacterial and fungal growth in the acidic environment of carbonated beverages. It is also used in salads, carbonated drinks, jams, fruit juices, and the pharmaceutical industry to keep liquid medicines fresh^12^. After oral, cutaneous, or inhalation exposure to SB, urticaria, asthma, rhinitis, or anaphylactic shock have been reported. Shortly after exposure, symptoms disappear within a few hours^7^.

Boric acid (BA, H_3_BO_3_, E284) is used in various human cosmetics and medicinal products, as well as antibacterial and antifungal products and veterinary products^13^. Boron-containing compounds are used in various consumer goods, most notably glass and ceramics, weatherproofing and fireproofing agents, fertilizers, and herbicides^14^. After oral, systemic, or cutaneous exposure, BA is hazardous to humans and animals at large concentrations^15^. Degenerative alterations in the liver, kidney, and brain were previously detected, and intracytoplasmic inclusion bodies in pancreatic acinar cells^16^.

Calcium propionate (CP, C_6_H_10_CaO_4_, E282) is an organic salt formed when calcium hydroxide and propionic acid react together^17^. CP is a powerful preservative with little or no flavor that works well against bacteria and mold and is extensively used in feeds, foods, and medications^18^. While CP consumption may not be toxic in high doses, its long-term exposure can have health consequences such as reported anxiety, visual hallucinations, hyperactivity, and sleep disorders^19^. Moreover, prolonged exposure to high levels of CP has been reported to evoke immunotoxic and hematotoxic impacts^20^.

According to the ages of consumers, localities, and estimation methodologies, the mean daily consumption of BHA ranges from 0.002 mg/kg/day to 0.3 mg/kg/day^21^. The concentrations of 25 mg PS/kg, 5 mg SB/kg^22^, and 1 mg CP/kg^23^ were reported as acceptable daily intakes of food preservatives. The Ministry of Agriculture of Turkey^24^ stated that BA can be used as a food preservative up to 4 g/l (4000 mg/l).

Food additives act as xenobiotics humans are exposed to^25^. The liver is responsible for a major portion of xenobiotic metabolism^26^. The kidney tubule cell produces proinflammatory cytokines that directly respond to infections or toxins^27^. These cells function as proinflammatory cells, or immune system responders are crucial in developing renal dysfunction^28^.

Toll-like receptors (TLRs) are important components of the hepatic immune system that play an vital role in liver physiology and pathology^29^. TLR-1, 2, 3, 4, and 6 are expressed by tubular epithelial cells in the kidney. TLR-4 is abundant in proximal and collecting tubules^30^. TLR-2 protein expression has also been present in various cell types in the kidney (tubules, medulla, glomeruli, and renal vasculature)^31^. Tumor necrosis factor-alpha (TNF-α) receptors and TLRs can activate nuclear factor kappa-light-chain-enhancer of activated B cells (NF-κB). This transcription factor largely enhances cell survival and proliferation^32^. The level of NF-κB significantly increased in response to the greater doses of food additives^33^. NF-κB is a key player in the TLR-4 signaling pathway and plays a role in the inflammatory response^34^.

In our earlier study, the 90-day oral dosing of the five food preservatives (PS, BHA, BA, SB, and CP) significantly altered the innate and humoral immune components^20^. Yet, very few studies explored oxidative damage and inflammation induction in liver and kidney organs after long exposure to PS, BHA, BA, SB, and CP. The current study evaluated and compared some toxic effects and metabolic alterations generated by PS, BHA, BA, SB, and CP. Also, the current work investigated histopathological changes in liver and kidney tissues following food preservatives exposure. Moreover, mRNA levels of hepatic and renal TLR-2 and 4, NF-κB, and TNF-α were studied using real-time quantitative polymerase chain reaction (RT-PCR).

## Materials and methods

### Tested chemicals

PS, BHA, BA, SB, and CP were attained from Sigma (St. Louis, MO, USA). All of the other chemicals and reagents were of analytical grade.

### Animals housing and grouping

Adult male albino rats (n = 60, average weight 160 ± 5 g) were bought from the National Research Center's breeding section (Giza, Egypt). All rats were kept in clean, well-ventilated steel mesh cages in a controlled environment (50–60% relative humidity, 20–24 °C, 12-h light–dark cycle). Throughout the experiment, rats had unlimited access to tap water and normal rodent food. Rats were acclimatized to the experimental circumstances for two weeks before being studied.

For 90 days, rats were weighed and arbitrarily assigned to one of six groups (n = 10) that were orally given distilled water, 4.5 mg PS /kg b.wt^22^, 0.09 mg BHA /kg b.wt^21^, 0.16 mg BA /kg b.wt^24^, 0.9 SB mg/kg b.wt^22^, or 0.18 mg CP /kg b.wt^23^. All food preservatives were consumed orally via orogastric gavage between 8 a.m. and 10 a.m. Every week, the amount of food preservatives acquired was modified based on the rats' body weight fluctuations. Pain, discomfort, damage, abnormal behavior, distress, breathing patterns, mucous membrane color, morbidity, and mortality were all closely monitored during the trial.

### Ethics declaration

The current animal experiment was carried out in line with the National Institutes of Health's general criteria for the care and use of laboratory animals in scientific investigations and were approved by Cairo University's research committee on the ethics of animal use (Approval number: VETCU01122022573). Furthermore, the study was carried out in line with the ARRIVE guidelines^35^.

### Sample collection

At the end of the trial (day 90), the rats fasted overnight. Then, the rats were weighed and anesthetized with an intramuscular injection of ketamine hydrochloride (50 mg/kg b.wt) and xylazine (5 mg/kg b.wt). All rats' blood was obtained in tubes without anticoagulants from the medial canthus. The tubes were left at room temperature for 20 min before centrifuging at 322 g for 10 min. The serum was carefully separated and stored at − 20 °C until biochemical analysis. Rats were then euthanized by cervical dislocation. During the autopsy, the liver and kidneys of rats were removed. The liver and kidney tissues were divided into three parts. The first was immediately transferred and stored in liquid nitrogen at − 80 °C for real-time PCR analysis with a 1 ml RNA ladder. The second liver and kidney samples were dissected and stored at − 20 °C to prepare homogenate for estimating oxidative stress biomarkers. Homogenization was accomplished in a cold solution of 0.015 M NaPOH buffer and 0.15 M NaCl (1:6 w/v; pH 7.8) using an Ultra-Turrax homogenizer. The last parts of the liver and kidney tissues were fixed with a 10% buffered formalin solution for histopathological examination.

### Hepatic and renal damage indicators assessment

A semi-automated Photometer (5010 V5+, RIELE GmbH & Co, Berlin, Germany) was utilized to evaluate hepatic and renal damage indicators from separated serum samples. The activities of alanine transaminase (ALT) and aspartate transaminase (AST) were measured by Bergmeyer, et al.^36^ method. The serum alkaline phosphatase (ALP) activity was measured using the method described by Kind and King^37^. According to Coulombe and Favreau^38^ and Fossati, et al.^39^, urea and creatinine levels were measured, respectively. The uric acid level was assessed following Barham and Trinder^40^ protocol. All biochemical variables were evaluated using commercial kits (BioMed Diagnostic Co., Cairo, Egypt).

### Evaluation of oxidative stress indicators in the liver and kidneys

Catalase (CAT), superoxide dismutase (SOD), and reduced glutathione (GSH) levels in liver and kidney homogenates were determined spectrophotometrically using Bio-diagnostic Co., Egypt kits reagents, according to the procedures of Sinha^41^, Nishikimi, et al.^42^, and Beutler, et al.^43^, respectively. Malondialdehyde (MDA) concentration was determined via a colorimetric assay according to the protocol of Ohkawa, et al.^44^.

### Analysis of TLR- 4, TLR-2, NF-κB and TNF-α gene expression

Total RNA was extracted from hepatic and renal tissue samples using the TRIzol reagent, as directed by the manufacturer (Invitrogen, Carlsbad, CA, USA). TaKaRa Bio Inc. provided instructions for real-time quantitative PCR utilizing SYBR-green. Table 1 shows the real-time PCR thermal outline and primer sequence, with an initial denaturation phase of 10 min at 95 °C and a final extension step of 10 min at 72 °C. Following normalization to β-actin, each transcript was quantified in three replicates by the 2^−ΔΔ^Ct technique of Livak and Schmittgen^45^ for the relative measurement of mRNA levels in tissue samples.

**Table 1:** Oligonucleotide primer sequences and real-time PCR conditions.

Gene name		Primer sequences	Reaction conditions
TLR4	F	5′-GTGAGCATTGATGAGTTCAG-3′	95 °C-15 s/59 °C-40 s/72 °C-40 s (40 cycles)
	R	3′-CATCTAATGATTGATAAGGATT-5′	
TLR2	F	5′-TCTGAGTTCCGTGACATAGG-3′	95 °C-15 s/59 °C-40 s/72 °C-40 s (40 cycles)
	R	3′-AGATGTAACGCAACAGATTC	
NF-κB	F	5′-GCTTTGCAAACCTGG GAATA-3′	95 °C-10 s/60 °C-30 s/72 °C-15 s (40 cycles)
	R	3′-CAAGGTCAGAAT GCACCAGA-5′	
TNF-α	F	5′-GGG GCC ACC ACG CTC TTC TGT-3′	95 °C-30 s/60 °C-30 s/72 °C-60 s (35 cycles)
	R	3′-GCA AAT CGGCTG ACG GTG TGG-5′	
β-actin	F	ACCACAGCTGAGAGGGAAATCG	94 °C-45 s/59 °C-44 s/72 °C-60 s (30 cycle)
	R	AGAGGTCTTTACGGATGTCAACG	

*F* forward primer, *R* reverse primer, *TLR4* toll-like receptors 4, *TLR2* toll-like receptors 2, *NF-kB* nuclear factor-kappa B, *TNF-α* tumor necrosis factor-alpha.

### Histopathological evaluation

Livers and kidneys were dissected from each animal, preserved in a 10% formalin solution for fixation, then dehydrated through ascending grades of alcohol, cleared in xylene, embedded, and blocked in paraffin. Three-micrometer thickness sections were stained with hematoxylin and eosin in line with the protocol of Suvarna, et al.^46^. Tissues were evaluated randomly with a light microscope (Olympus, Tokyo, Japan) by a pathologist who had no idea to which group the rat belonged. The microscopic scoring was graded on a scale of the absence of lesion (−) or mild ( +), moderate (++), and severe changes (+++), according to Arsad, et al.^47^.

### Data analysis

The Kolmogorov–Smirnov and Levene's tests were used to check the data for normality and variance homogeneity, respectively. When normality assumptions were met, using IBM SPSS Statistics, version 21 (IBM; Armonk, New York, USA)^48^, data were analyzed by one-way analysis of variance (ANOVA) to statistically define the variation between groups, followed by Tukey's multiple range post hoc test for pairwise comparisons. The data has been displayed as means ± SE for each group. At *P* < 0.05, mean differences were considered significant. Moreover, the GraphPad Prism version 8 (GraphPad Software, San Diego, CA, USA) was used for data presentation^49^. The principal component analysis was applied on the replicates of biochemical and molecular analyses by the Granato et al.^50^ method.

## Results

### Effect of BHA, PS, SB, BA, or CP on liver and kidney functions variables

The changes in hepatorenal function indicators in the rats exposed to BHA, PS, SB, BA, or CP for 90 days are displayed in Table 2. Serum AST and ALT levels were significantly increased by 35%, 134%, 123%, 73%, and 83% and by 146%, 99%, 234%, 1607%, and 73% in the BHA, PS, SB, BA, and CP groups, respectively, compared to the control group. Serum ALP activity was significantly increased by 136% and 690% in the BHA and BA groups, respectively. It showed a significant decrease of 60%, 63%, and 61% in PS, SB, and CP groups, respectively, compared to the control group.

**Table 2:** Serum biochemical analysis and oxidative stress indices of hepatic and renal tissues of butylated hydroxyl anisole (BHA), potassium sorbate (PS), sodium benzoate (SB), boric acid (BA), or calcium propionate (CP)-treated rats.

Parameter	Treatment					
	Control	BHA	PS	SB	BA	CP
AST (U/L)	85.88^f^ ± 2.56	116.13^e^ ± 2.27	201.30^a^ ± 0.51	191.61^b^ ± 1.45	148.33^d^ ± 1.65	157.07^c^ ± 2.59
ALT (U/L)	20.72^e^ ± 0.72	51.06^c^ ± 2.02	41.20^d^ ± 0.53	69.14^b^ ± 2.23	353.63^a^ ± 2.74	35.87^d^ ± 2.28
ALP (U/L)	131.57^c^ ± 2.39	309.90^b^ ± 5.31	52.37^d^ ± 2.53	48.80^d^ ± 0.78	1039.33^a^ ± 0.62	51.90^d^ ± 1.08
Urea (mg/dL)	32.07^e^ ± 0.81	104.03^b^ ± 2.72	76.13^d^ ± 2.88	139.33^a^ ± 3.01	87.67^c^ ± 2.87	92.00^c^ ± 1.63
Creatinine (mg/dL)	1.12^d^ ± 0.01	1.24^c^ ± 0.03	1.23^c^ ± 0.03	1.22^c^ ± 0.01	1.39^b^ ± 0.04	1.67^a^ ± 0.01
Uric acid (mg/dL)	2.30^d^ ± 0.08	2.66^cd^ ± 0.05	2.53^cd^ ± 0.25	2.89^c^ ± 0.07	3.34^b^ ± 0.11	4.19^a^ ± 0.07
**Hepatic tissues**						
MDA (μmol/L)	13.22^c^ ± 1.29	14.83^c^ ± 2.65	26.77^b^ ± 4.21	27.52^ab^ ± 3.44	31.22^a^ ± 4.89	15.14^c^ ± 3.6
SOD (µg/mg protein)	95.3^a^ ± 5.2	87.54^b^ ± 4.85	57.45^d^ ± 4.46	58.18^d^ ± 6.18	51.42^e^ ± 4.81	79.42^c^ ± 3.66
CAT (μmol H_2_O_2_ decomposed/g tissue)	6.44^a^ ± 0.82	5.98^a^ ± 0.85	2.74^c^ ± 0.48	2.94^c^ ± 0.73	2.05^d^ ± 0.65	4.92^b^ ± 0.45
GSH (mg/g tissue)	28.45^a^ ± 2.39	27.66^ab^ ± 3.14	13.92^c^ ± 2.42	14.11^c^ ± 2.74	12.34^c^ ± 2.8	22.18^b^ ± 2.62
**Renal tissues**						
MDA (μmol/L)	9.52^c^ ± 0.43	12.26^bc^ ± 3.96	22.62^a^ ± 3.62	21.75^a^ ± 3.85	25.46^a^ ± 5.31	14.25^b^ ± 2.42
SOD (µg/mg protein)	85.3^a^ ± 3.4	81.54^a^ ± 5.25	55.64^c^ ± 3.54	59.21^c^ ± 5.22	48.66^d^ ± 3.22	72.32^b^ ± 2.58
CAT (μmol H_2_O_2_ decomposed/g tissue)	4.42^a^ ± 0.32	4.27^a^ ± 0.28	1.95^c^ ± 0.26	2.12^c^ ± 0.17	1.82^d^ ± 0.38	3.52^b^ ± 0.24
GSH (mg/g tissue)	23.2^a^ ± 2.62	20.95^ab^ ± 2.28	10.53^c^ ± 2.85	11.44^c^ ± 2.82	9.26^c^ ± 3.4	19.26^b^ ± 2.15

Control group: received distilled water. BHA group: 0.09 mg/kg b.wt. PS group: received 4.5 mg/kg b.wt. SB group: received 0.9 mg/kg b.wt. BA group: received 0.16 mg/kg b.wt. CP group: received 0.18 mg/kg b.wt. Rats were orally given all food preservatives for 90 days.

*AST* aspartate transaminase, *ALT* alanine transaminase, *ALP* alkaline phosphatase, *MDA* malondialdehyde, *SOD* superoxide dismutase, *CAT* catalase, *GSH* reduced glutathione.

Means within same row carrying different superscripts are significantly different at *p* < 0.05. Values are shown as the mean ± SE. n = 5/group.

Compared to the control group, the BHA, PS, SB, BA, and CP groups showed a significant rise in serum urea levels (224%, 137%, 334%, 173%, and 187%, respectively) and serum creatinine (11%, 10%, 9%, 24%, and 49%, respectively). The SB, BA, and CP groups had significantly higher serum uric acid levels than the control group by 26%, 45%, and 82%, respectively (Table 2).

### Effect of BHA, PS, SB, BA, or CP on oxidative stress biomarkers

Table 2 shows the changes in hepatic oxidative stress indicators in rats continually treated with BHA, PS, SB, BA, or CP for 90 days. Hepatic SOD levels were significantly reduced by 8%, 40%, 39%, 46%, and 17% in the BHA, PS, SB, BA, and CP groups, respectively, compared with the control group. Hepatic CAT levels were significantly reduced by 57%, 54%, 68%, and 24% in the PS, SB, BA, and CP groups, respectively, compared to the control group. Furthermore, hepatic GSH levels were significantly reduced by 51%, 50%, 57%, and 22% in the PS, SB, BA, and CP groups, respectively, compared to the control group. Compared with the control group, the PS, SB, and BA groups showed a significant increase in hepatic MDA levels (102%, 108%, and 136%), respectively.

Concerning indicators of renal oxidative stress in the rats exposed to BHA, PS, SB, BA, or CP for 90 days, the renal SOD, CAT, and GSH levels were significantly reduced by 35%, 31%, 43%, and 15% and by 56%, 52%, 59%, and 20% and by 55%, 51%, 60% and 17% in the PS, SB, BA, and CP groups, respectively, compared with the control group. Compared to the control group, the PS, SB, BA, and CP groups all had significantly higher renal MDA levels than the control group (138%, 128%, 167%, and 50%, respectively) (Table 2).

### Effect of BHA, PS, SB, BA, or CP on hepatic and renal TLR-4 and TLR-2 gene expression

**Figure 1:**
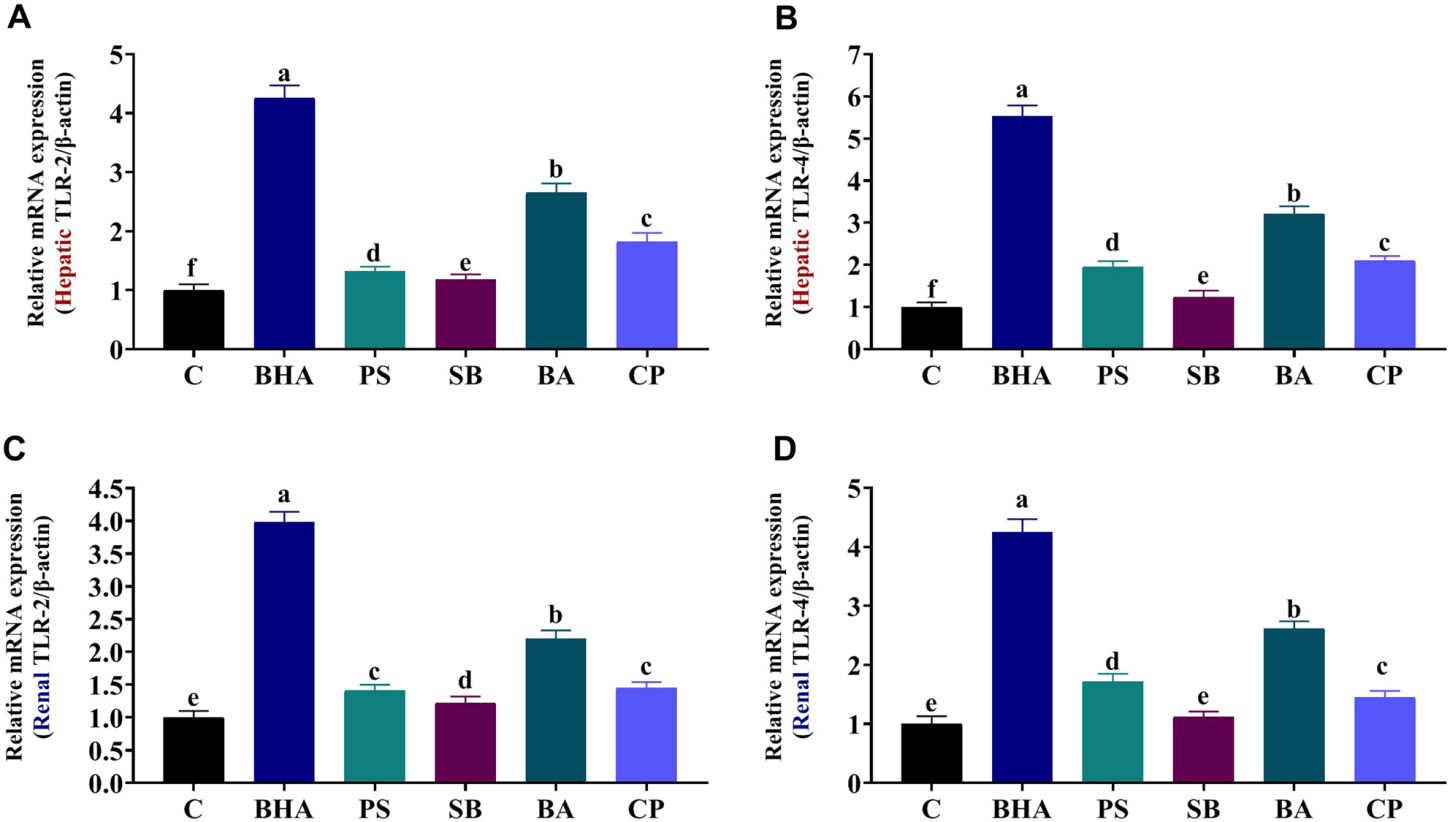
Expression of toll-like receptors 2 (TLR2) and toll-like receptors 4 (TLR4) in the hepatic and renal tissues of butylated hydroxyanisole (BHA), potassium sorbate (PS), sodium benzoate (SB), boric acid (BA), or calcium propionate (CP)-treated rats. β-Actin was used as the internal control. Data are expressed as the mean ± SE (n = 3 replicates). Columns with different superscripts are significantly different (one-way ANOVA, p < 0.05).

Figure 1A,B demonstrate the effects of BHA, PS, SB, BA, and CP oral dosage for 90 days on hepatic TLR-2 and TLR-4 mean mRNA expression levels. Hepatic TLR-4 expression was increased significantly by 5.52, 1.95, 1.24, 3.21, and 2.10 fold in the BHA, PS, SB, BA, and CP groups, respectively, relative to the control group. The mean expression of Hepatic TLR-2 rose by 4.26, 1.32, 1.18, 2.65, and 1.82 fold in the BHA, PS, SB, BA, and CP groups, respectively, compared to the control group.

In the BHA, PS, BA, and CP groups, renal TLR-4 expression was elevated considerably by 4.25, 1.72, 2.62, and 1.45 fold, respectively, relative to the control group. In the BHA, PS, SB, BA, and CP groups, mean renal TLR-2 expression was elevated by 3.98, 1.41, 1.22, 2.20, and 1.45 fold, respectively, compared to the control group (Fig. 1C,D).

### Effect of BHA, PS, SB, BA, or CP on hepatic and renal NF-κB and TNF-α gene expression

**Figure 2:**
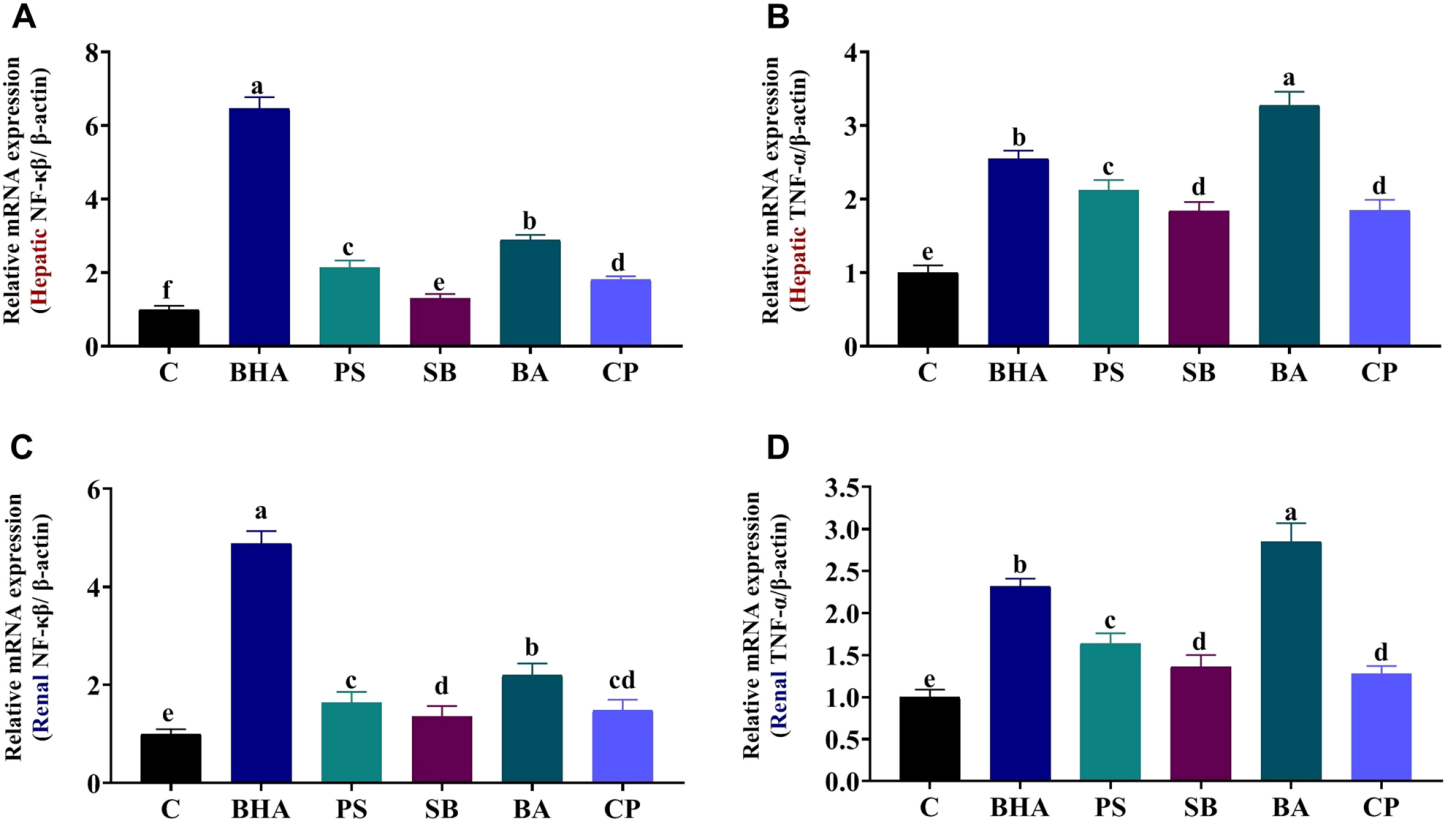
Expression of nuclear factor-kappa B (NF-κB) and tumor necrosis factor-alpha (TNF-α) in the hepatic and renal tissues of butylated hydroxyanisole (BHA), potassium sorbate (PS), sodium benzoate (SB), boric acid (BA), or calcium propionate (CP)- treated rats. β-Actin was used as the internal control. Data are expressed as the mean ± SE (n = 3 replicates). Columns with different superscripts are significantly different (one-way ANOVA, p < 0.05).

In the liver, NF-κB expression was significantly upregulated by 6.46, 2.14, 1.31, 2.89, and 1.81 fold in response to BHA, PS, SB, BA, and CP exposure, respectively, relative to the control. TNF-α expression was also elevated considerably by 2.55, 2.12, 1.84, 3.28, and 1.85 fold in the BHA, PS, SB, BA, and CP groups, respectively, compared to the control group (Fig. 2A,B). While renal NF-κB and TNF-α expression were significantly upregulated by 4.89, 1.64, 1.36, 2.20, and 1.48 fold and by 2.32, 1.64, 1.36, 2.85, and 1.28 fold in response to BHA, PS, SB, BA, and CP exposure, respectively, compared to the control (Fig. 2C,D).

### Effect of BHA, PS, SB, BA, or CP on histopathological findings of liver

**Figure 3:**
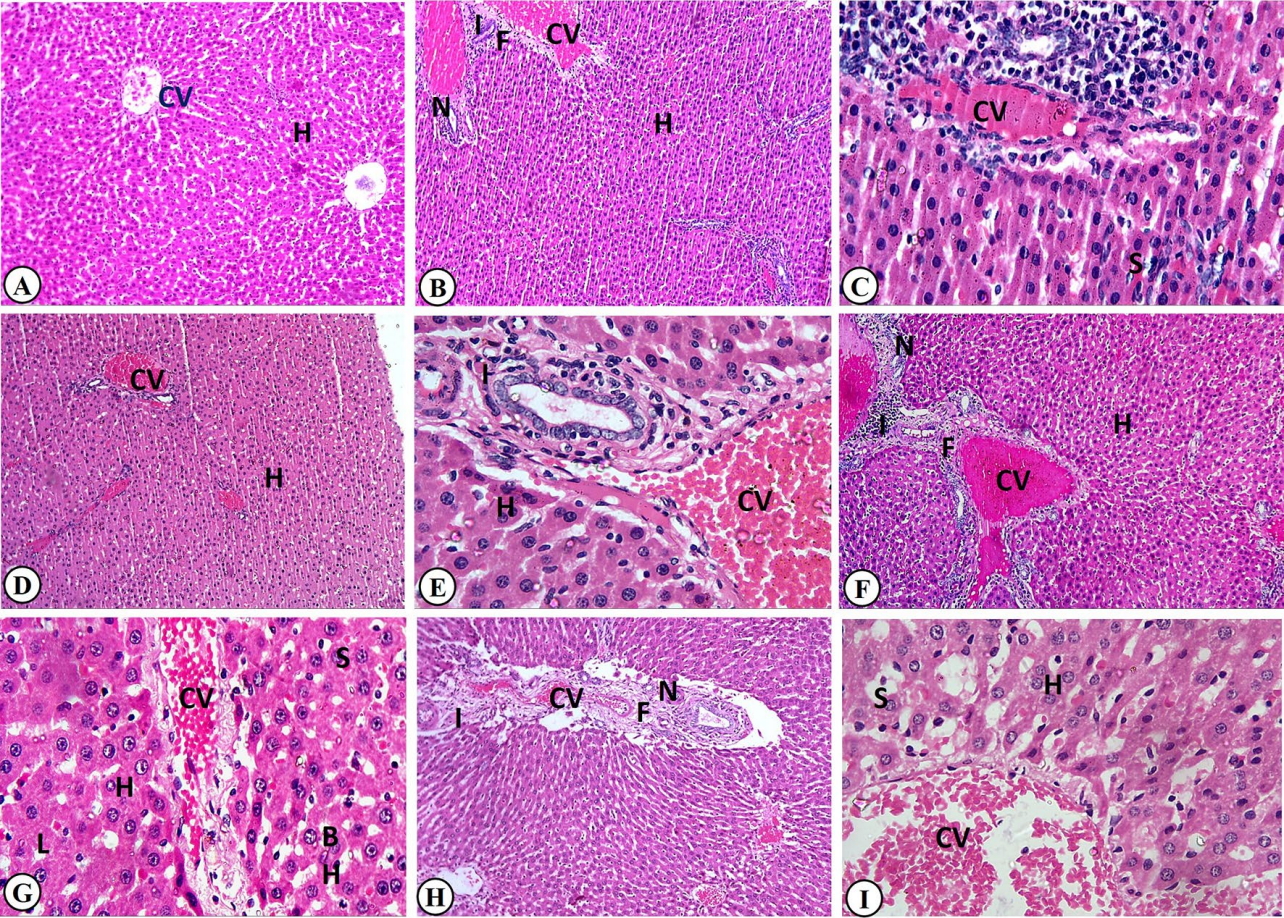
Representative histopathological photomicrographs of hematoxylin-stained cross-sections of rat livers. (**A**) Control group: showing normal architecture with normal hepatocytes in the hepatic cords (H) and central vein (CV) (H&E stain, × 100). Butylated hydroxyanisole-treated group: (**B**) showing hepatocytes disorganization (H), dilated and congested central vein (CV), inflammatory cell infiltrations (I) in the portal area, dilated bile ductules (N), and periportal fibrosis (F) (H&E stain × 100). (**C**) showing dilated and congested central vein (CV) and sinusoids (S), inflammatory cell infiltrations (I) in the portal area, and bile duct hyperplasia (B) (H&E stain, × 400). Potassium sorbate-treated group (**D**): showing hepatocyte disorganization (H) and congested central vein (CV) (H&E stain × 40). (**E**) showing hepatocytes degenerative changes (H) with pyknotic nuclei, congested central vein (CV), dilated blood sinusoids, and inflammatory cell infiltrations (I) in the portal area (H&E stain, × 400). Sodium benzoate-treated group showing: (**F**) hepatocytes disorganized cords (H), congested central vein (CV), mononuclear leucocytic infiltrations (I), newly formed bile ductules (N), and fibrosis (F) in the portal area (H&E stain, × 100). (**G**) Periportal degenerative changes of hepatocytes (H) with pyknotic nuclei, nuclear loss (L) or binucleated hepatocytes (BH), dilated and congested central vein (CV), and hepatic sinusoids dilatation (S) (H&E stain, × 400). Boric acid-treated group showing: (**H**) congested portal vein (CV), inflammatory cell infiltrations (I) in the portal area, newly formed bile ductules (N), and portal fibrosis (F) (H&E stain, × 100). Calcium propionate-treated group showing: (**I**) congested portal vein (CV) and hepatic sinusoids(S) as well as degenerative changes of hepatocytes (H) with pyknotic nuclei (H&E stain, × 400).

The macroscopic appearance of the liver of the control group showed a normal appearance, while the treated groups showed hepatomegaly and congestion. Microscopically, the normal control group showed normal architecture of liver tissue with clearly defined hepatocytes around the central vein and well-arranged hepatic cords between sinusoids and normal bile ducts (Fig. 3A). While the treated groups revealed that the normal lobular pattern of hepatic cords was distorted, resulting in the dilatation of some sinusoids and obliteration of others. Periportal vacuolar degenerative changes of hepatocytes with pyknotic nuclei and Kupffer cell proliferation were noted. There was nuclear loss or binucleated hepatocytes. Focal and/or diffuse mononuclear inflammatory cell infiltrations, mainly lymphocytes and macrophages, were detected in the portal area. The central vein and hepatic sinusoids were dilated and congested, and lymphatic vessel dilatation was observed. Interlobular edema was also noticed. Bile duct hyperplasia and epithelial destruction with newly formed bile ductules were observed and associated with periportal fibrosis. The severity of these pathological alterations differed according to the treated material among the different groups, as mentioned in Table 3. They became more pronounced in BA (Fig. 3H), BHA (Fig. 3B,C), and SB (Fig. 3F,G) treated groups, followed by PS (Fig. 3D,E) and, then CP (Fig. 3I) treated groups.

**Table 3:** The lesion scoring of hepatic tissues of butylated hydroxyl anisole (BHA), potassium sorbate (PS), sodium benzoate (SB), boric acid (BA), or calcium propionate (CP)-treated rats.

Parameter	Treatment					
	Control	BHA	PS	SB	BA	CP
A. Macroscopic appearance						
1. Hepatomegaly	**−**	**+++**	**++**	**+++**	**++**	**++**
2. Congestion	**−**	**+++**	**++**	**+++**	**+**	**++**
B. Microscopic appearance						
1. Hepatic cords disorganization	**−**	**+++**	**++**	**++**	**++**	**++**
2. Degenerative changes	**−**	**+++**	**+**	**++**	**+++**	**++**
3. Inflammatory cell infiltrations	**−**	**+++**	**++**	**++**	**+++**	**+**
4. Blood vessels congestion and dilatation	**−**	**+++**	**+++**	**+++**	**+**	**++**
5. Bile duct hyperplasia	**−**	**++**	**++**	**++**	**++**	**+**
6. Periportal fibrosis	**−**	**++**	**+**	**++**	**+**	**+**

Control group: received distilled water. BHA group: 0.09 mg/kg b.wt. PS group: received 4.5 mg/kg b.wt. SB group: received 0.9 mg/kg b.wt. BA group: received 0.16 mg/kg b.wt. CP group: received 0.18 mg/kg b.wt. Rats were orally given all food preservatives for 90 days.

−: Absence of lesion; +: Mild; ++: Moderate; +++: Severe damage. N = 10/each group.

### Effect of BHA, PS, SB, BA, or CP on histopathological findings of kidney

**Figure 4:**
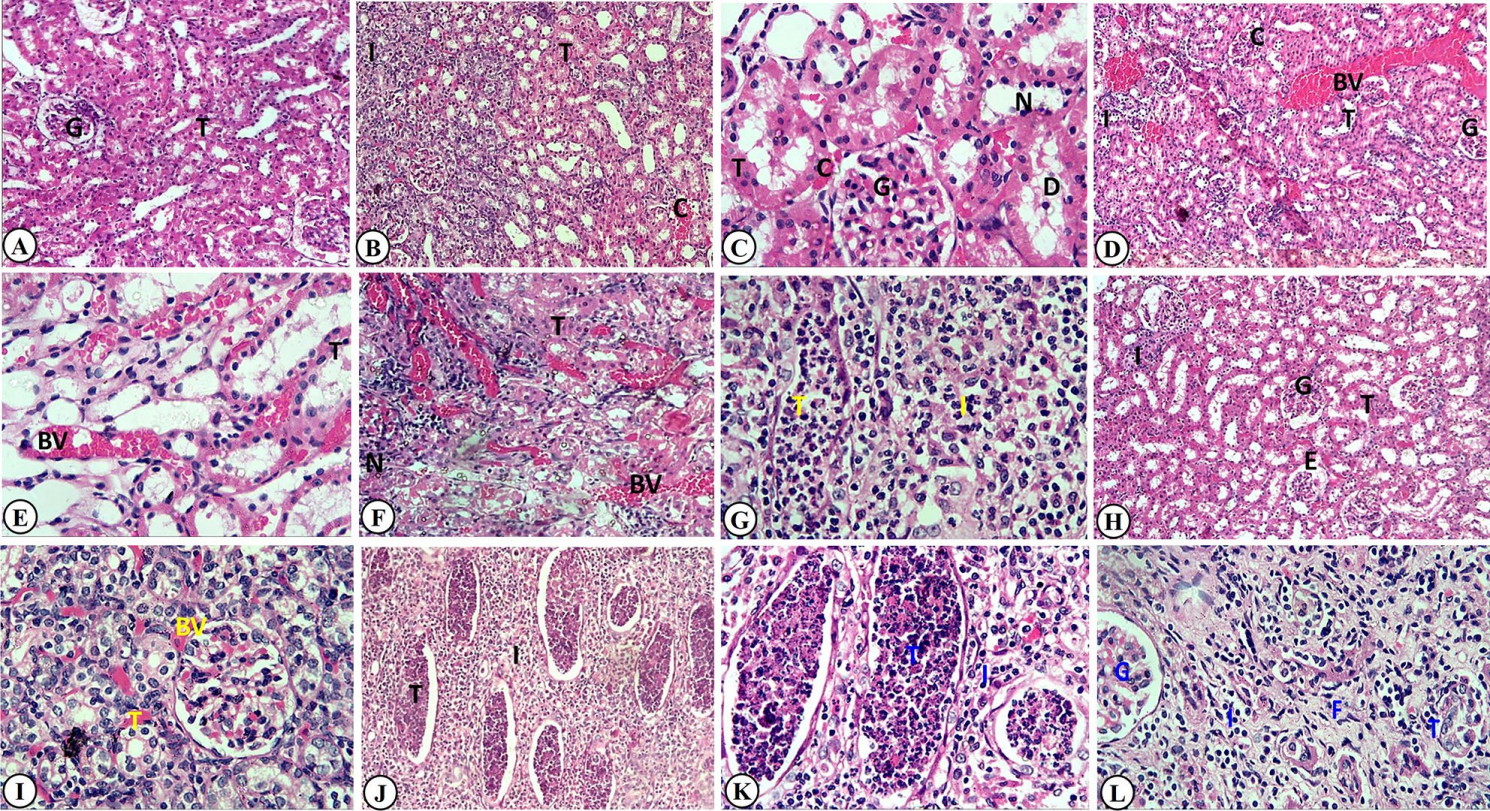
Representative histopathological photomicrographs of hematoxylin-stained cross-sections of rat kidneys. (**A**) Control group: showing normal structure with renal glomeruli (G) and tubules (T) (H&E stain, × 100). Butylated hydroxyanisole-treated group showing: (**B**) degenerative changes in renal tubular cells (T), focal leucocytic infiltrations (I), and congestion of intertubular blood vessels (C) (H&E stain × 100). (**C**) Granular degeneration in renal tubular cells (T), the desquamated epithelium (D), cellular swelling with pyknotic nuclei (N), congestion of intertubular blood vessels (C) with vasculitis (V) (H&E stain, × 400). Potassium sorbate-treated group showing: (**D**) degeneration of renal tubules (T), intertubular blood vessels (BV) and glomerular tuft (C) congestion, focal interstitial nephritis(I), glomerular shrinkage (G) (H&E stain, × 100). (**E**) Tubular granular degeneration (T) and congestion of intertubular blood vessels (BV) (H&E stain, × 400). Sodium benzoate-treated group showing: (**F**) chronic interstitial glomerulonephritis (N) with cloudy swelling of tubules (T) and congestion of intertubular blood vessels (BV) (H&E stain × 100). (**G**) chronic interstitial nephritis with vacuolated cytoplasm and pyknotic nuclei, degenerate and desquamated epithelial cells, and leucocytic infiltrations mainly neutrophils within tubules (T) as well as interstitial inflammatory cell infiltrates (I) (H&E stain, × 400). Boric acid-treated group showing: (**H**) tubular cell swelling (T), congestion of intertubular blood vessels and glomerular tuft (G), interstitial leucocytic infiltrations (I) and glomerular edema (E), and shrinkage (H&E stain, × 100). (**I**) Vacuolar degeneration in renal tubular cells (T), desquamation of lining epithelium, narrowing of tubular lumen, and congestion of intertubular blood vessels (BV) (H&E stain, × 400). Calcium propionate-treated group showing: (**J**) chronic interstitial glomerulonephritis with dilated tubules and filled with degenerated cells, leucocytic infiltrations, and desquamated epithelial cells (T), interstitial inflammatory cell infiltrates (I) (H&E stain, × 100). (**K**) Degenerated cells, leucocytic infiltrations, desquamated epithelial cells within dilated tubules (T), interstitial inflammatory cell infiltrates (I), and tubular atrophy (H&E stain, × 400). (**L**) Chronic interstitial glomerulonephritis with atrophy of tubules (T), interstitial fibrosis (F), and inflammatory cell infiltrates (I) as well as glomerular edema, hyperemia, and shrinkage (G) (H&E stain, × 400).

Grossly, the kidneys of the control group showed no gross abnormalities, while those of BHA, PS, and BA treatment appeared congested and enlarged and rat kidneys treated with SB and CP were small and pale. The histological examination showed no histoarchitecture alterations with normal cortex and medulla. There was a well-developed glomerulus, Bowman's capsule, and proximal and distal convoluted tubules in the kidneys of the control group (Fig. 4A). As shown in Fig. 4, in the animals that received BHA (Fig. 4B,C), PS (Fig. 4D,E) and BA (Fig. 4H,I) treatment, kidney tissue sections showed marked granular degeneration in renal tubular cells. There was cellular swelling with indistinct cell borders and pyknotic nuclei. Desquamation of lining epithelium of renal tubules was observed. There was eosinophilic protein cast in some renal tubules' lumen, narrowing the tubule lumen while dilating others. Moderate shrinkage of glomeruli with slightly dilated Bowman's space, thickening of the glomerular basement membrane, and glomerular tuft and hemorrhage congestion was seen. Focal interstitial nephritis with leucocytic infiltrations was noted. Also, there was congestion of intertubular blood vessels with vasculitis and interstitial edema.

On the other side, the rat's kidney treated with SB (Fig. 4F,G) and CP (Fig. 4J–L) was characterized by remarkable chronic interstitial glomerulonephritis with severe degenerative changes. Both proximal and distal convoluted tubules were dilated with desquamated and degenerated epithelial cells and leucocytic infiltrations, mainly neutrophils. Granular degeneration was increased in some tubules, while others appeared highly swollen and dissociated with vacuolated cytoplasm, indistinct cell borders and pyknotic nuclei. Multifocal and/or diffuse interstitial fibrosis and inflammatory cell infiltration with variable numbers of lymphocytes, macrophages, histiocytes, plasma cells, and few neutrophils were the histological hallmarks of both groups. Tubular atrophy was detected with a prominently thickened, wrinkled basement membrane and a narrow or no lumen. Also, other tubules frequently underwent compensatory dilated lumen and formation of the hyaline cast. Furthermore, the renal corpuscle was also damaged, including glomerular edema, thickening of the basement, and shrinked glomeruli. There was congestion of the glomerular tuft and intertubular blood vessels associated with vasculitis. The severity of the previous renal damages differed among different groups, as mentioned in Table 4.

**Table 4:** The lesion scoring of renal tissues of butylated hydroxyl anisole (BHA), potassium sorbate (PS), sodium benzoate (SB), boric acid (BA), or calcium propionate (CP)-treated rats.

Parameter	Treatment					
	Control	BHA	PS	SB	BA	CP
A. Macroscopic appearance						
1. Congestion and enlargement	**−**	**++**	**++**	**−**	**+**	**−**
2. Small size and paleness	**−**	**−**	**−**	**++**	**−**	**+++**
B. Microscopic appearance						
1. Degenerative renal tubules changes	**−**	**++**	**+++**	**+++**	**+++**	**+++**
2. Tubular atrophy	**−**	**+**	**+**	**+++**	**++**	**+++**
3. Dilated tubules with degenerated cells and leucocytic infiltrations	**−**	**−**	**−**	**++**	**−**	**+++**
4. Hyaline cast	**−**	**+**	**+**	**+++**	**+**	**+++**
5. Shrinkage of glomeruli	**−**	**+**	**++**	**+++**	**++**	**+++**
6. Congestion of glomerular tuft	**−**	**++**	**++**	**+++**	**++**	**+**
7. Interstitial nephritis	**−**	**++**	**+**	**+++**	**+**	**+++**
8. Interstitial fibrosis	**−**	**−**	**−**	**+**	**−**	**+++**
9. Interstitial b.v. Congestion	**−**	**+++**	**+++**	**+++**	**+++**	**+**
10. Edema	**−**	**++**	**++**	**+**	**++**	**−**

Control group: received distilled water. BHA group: 0.09 mg/kg b.wt. PS group: received 4.5 mg/kg b.wt. SB group: received 0.9 mg/kg b.wt. BA group: received 0.16 mg/kg b.wt. CP group: received 0.18 mg/kg b.wt. Rats were orally given all food preservatives for 90 days.

−: Absence of lesion; +: Mild; ++: Moderate; +++: Severe damage. N = 10/each group.

### Correlation analysis of analyzed indicators in hepatic and renal tissues

**Figure 5:**
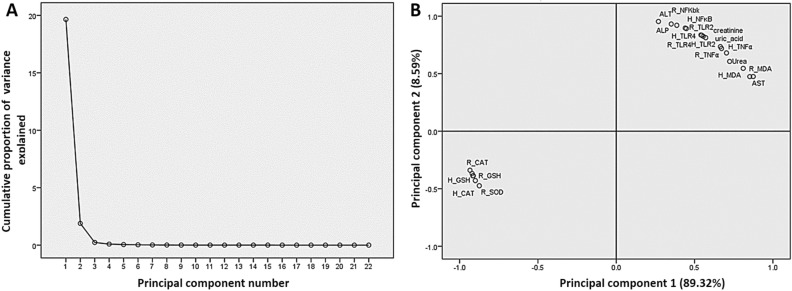
The principal component analysis plot shows the estimated variables' relationships. (**A**) Cumulative proportion of variance as a function of the number of principal components (PC). (**B**) All biochemical and gene expressions indicators are plotted as a function of PC1 and PC2, which account for 89.32% and 8.59% of the variance, respectively. *H_* hepatic, *R_* renal, *AST* aspartate transaminase, *ALT* alanine transaminase, *ALP* alkaline phosphatase, *MDA* malondialdehyde, *SOD* superoxide dismutase, *CAT* catalase, *GSH* reduced glutathione, *NF-κB* nuclear factor kappa-light-chain-enhancer of activated B cells, *TLR-2* toll-like receptors 2, *TLR-4* toll-like receptors 4, *TNF-α* tumor necrosis factor-alpha.

Principal components analysis was used to test the links between the liver and kidney function serum parameters and oxidative stress and lipid indicators and TLR-4, TLR-2, NF-κB, and TNF-α gene expressions in hepatic and renal tissues. The loading plot of the first two components was plotted, as displayed in Fig. 5, and both components represented about 97.91% of the overall variation in the experimental data. In the loading plot, the closely associated variables (< 90°) have strong correlations and are positively correlated with one another. Accordingly, the variables including liver enzymes (ALT, AST, and ALP), renal damage products (urea, uric acid, and creatinine), and lipid peroxidation marker (MDA) and TLR-4, TLR-2, NF-κB, and TNF-α gene expressions in hepatic and renal tissues are grouped together and highly correlated with the first component. Also, the variables of hepatic and renal antioxidants including GSH, SOD, and CAT were grouped together and correlated with the second component. The liver enzymes, renal damage products, and lipid peroxidation and TLR-4, TLR-2, NF-κB, and TNF-α gene expressions in hepatic and renal tissues were highly negatively correlated with the hepatic and renal antioxidants contents.

## Discussion

The liver is the main site of xenobiotic metabolism and is consequently highly susceptible to its harmful impacts^27,51^. In the current study, a marked impaired liver function was detected in the rats orally given BHA, PS, SB, BA, and CP for 90 days, which reflected in a significant increase in serum AST and ALT levels than control ones. These findings could be related to the pathological perturbations induced by the tested feed additives in hepatic tissues, including vacuolar degeneration of hepatocytes with pyknotic nuclei and Kupffer cell growth. When the hepatocyte membrane is broken, cytosolic enzymes are released into the bloodstream^52^. Also, the increased AST and ALT enzyme activity could be explained by the generation of free radicals, especially in the PS, SB, BA, and CP-treated groups. These free radicals react with polyunsaturated fatty acids in the cell membrane, causing mitochondrial and plasma membrane damage and enzyme leakage^53^. Similar findings were reported by Mehedi et al.^54^.

The BHA and BA-exposed rats had a considerably increased ALP level. In contrast, the PS, SB, and CP groups showed a significant reduction in ALP levels compared to the control groups. The increase in ALP concentration may be attributed to the cytotoxic effect of BHA and BA, which led to damage to liver cells^26^. On the other hand, the inhibitory effect of PS, SB, and CP on ALP enzyme activity might be related to their interaction with Zn as ALP is a Zn^2+^-metalloprotein. Monanu et al.^55^ correlated the SB inhibitory action on ALP enzyme to its aggressive processing in the liver at chronic levels that may burden the liver, resulting in liver malfunction.

The kidney is highly susceptible to developing various forms of injury because of its role as the main excretory route of most xenobiotics and toxins^56^. Changes in the tubular reabsorption threshold, renal blood flow, and glomerular filtration rate could be blamed for these problems^26^. The serum urea and creatinine levels are commonly measured to assess kidney function^57,58^. Herein, a substantial increase in serum urea, creatinine, and uric acid was recorded in the rats orally given BHA, PS, SB, BA, and CP for 90 days. The increased serum renal damage products were also concomitant with various histopathological alterations in renal tissues of food preservatives-exposed rats, including granular degeneration in renal tubular cells, moderate shrinkage of glomeruli, congestion of intertubular blood vessels, and interstitial edema. Tawfek, et al.^26^ previously reported similar findings in rats exposed to other food additives like monosodium glutamate, tartrazine, and sunset yellow. This could be due to the detected oxidative stress and inflammation in the renal tissues resulting from the tested food preservatives, as revealed by the biochemical, molecular, and histopathological findings. Besides, BHA, PS, SB, BA, and CP could have interfered with creatinine metabolism, resulting in increased synthesis, or affected all or part of the tissues' tubular excretion functional ability^59,60^.

Oxidative stress has been suggested as an underlying mechanism for many food preservative toxicity^61,62^. In the current experiment, orally given PS, SB, BA, and CP rats showed a substantial drop in hepatic and renal SOD, CAT, and GSH levels. The earlier antioxidant depletion may be due to their excessive utilization in inactivating the free radicals generated by the tested food additives^61^. These observations were in line with the study of Yetuk et al.^63^, which reported a decrease in SOD, CAT, glutathione peroxidase, and GST activities in the erythrocytes by SB. Additionally, the enhanced lipid peroxidation recorded in the current work could be linked to the direct effect of increased ROS formation caused by PS, SB, BA, and CP administration. Comparably, other food additives like monosodium glutamate, tartrazine, and sunset yellow induced oxidative stress and lipid peroxidation in the renal tissues of rats^26^. Moreover, BA has been reported to cause oxidative stress and lipid peroxidation in various tissues^64,65^. Furthermore, the study by Khodaei, et al.^66^ reported a significant rise in lipid peroxidation, decreased GSH content, and increased CAT activity in the kidney tissues of SB-exposed mice. On the other hand, a non-significant change in GSH, CAT, SOD, and MDA in liver and kidney tissues, but a significant decrease in hepatic SOD was found in rats that consumed BHA compared to the control group. In this regard, BHA might increase antioxidant enzymes synthesis as a compensatory mechanism to prevent kidney and liver damage^67^. Moreover, the aromatic ring contained in BHA can sequester free-radical ROS, allowing them to be stabilized^68^. Indeed, this molecule acts as a ROS scavenger by giving labile hydrogen to oxygen radicals formed by fatty acids, resulting in an oxidized phenolic ion that is stabilized by the benzene ring's resonance^69^.

**Figure 6:**
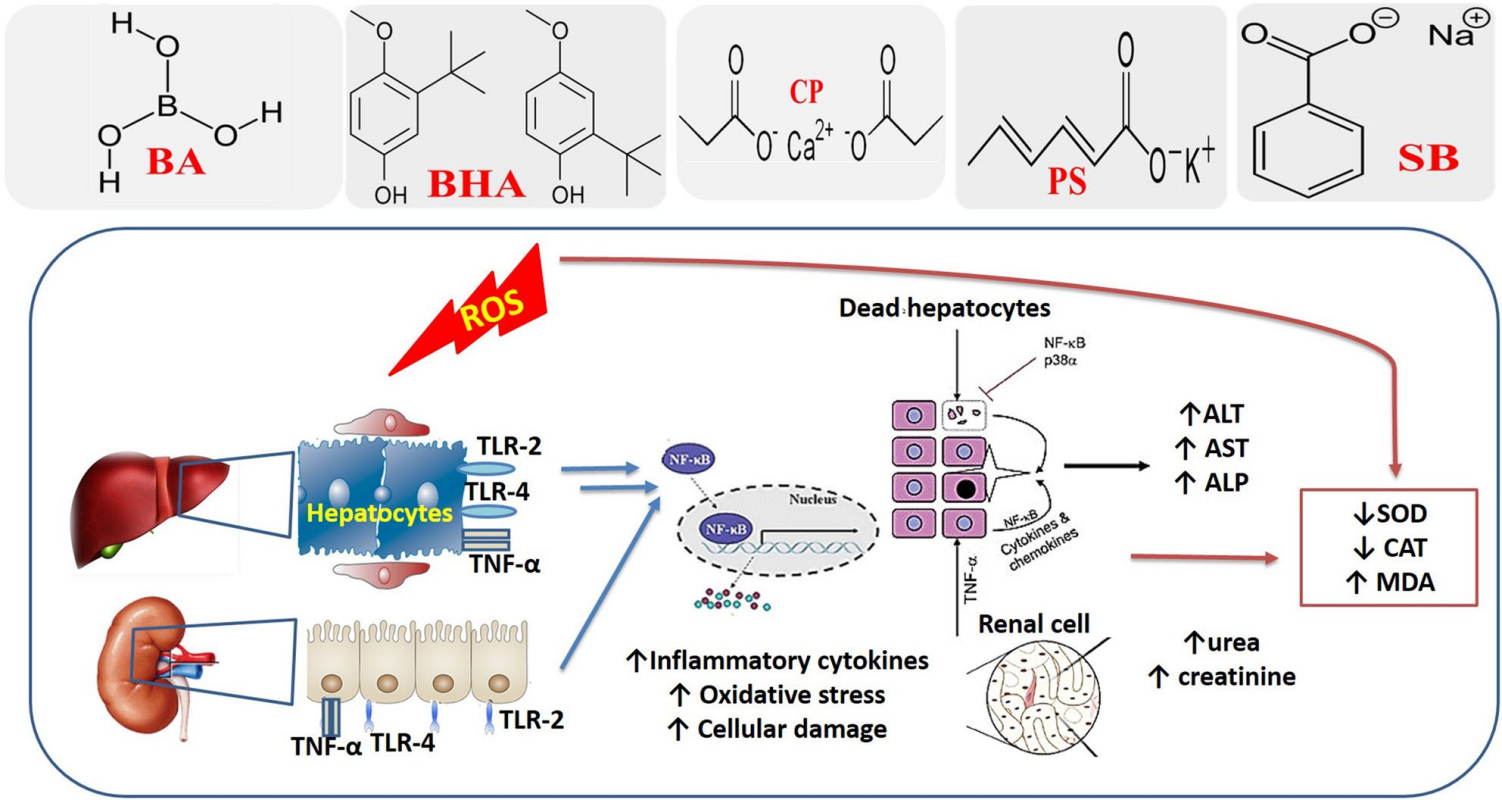
Schematic presentation of the proposed mechanism of hepatorenal damage induced by potassium sorbate (PS), butylated hydroxyanisole (BHA), sodium benzoate (SB), calcium propionate (CP), and boric acid (BA). *ALP* alkaline phosphatase, *ALT* alanine transaminase, *AST* aspartate transaminase, *CAT* catalase, *CP* calcium propionate, *GSH* reduced glutathione, *MDA* malondialdehyde, *NF-κB* nuclear factor kappa-light-chain-enhancer of activated B cells, *SOD* superoxide dismutase, *TLR-2* toll-like receptors 2, *TLR-4* toll-like receptors 4, *TNF-α* tumor necrosis factor-alpha.

Oxidative stress can activate various transcription factors, which results in the differential expression of some genes implicated in inflammatory pathways^70^. In the current experiment, TLR-4, TLR-2, NF-κB, and TNF-α genes mRNA expressions in hepatic and renal tissues were assessed. TLR-2 and TLR4 are expressed in all hepatic cells and plays a role in tissue damage induced by various causes^71^. Moreover, TLR-2 and TLR-4 plays an imperative role in the pathophysiology of acute kidney injury and may be a potential therapeutic target to lessen renal damage in response to various pathological stimuli^72,73^. In the kidney, accumulation of ROS and reactive nitrogen species induce renal dysfunction^74^ as well as increased TLR- 4 expressions by tubular cells^75^. The stimulation of the TLR-2 and TLR4 signal pathway initiates a series of events that include the transfer of NF-κB to the nucleus, stimulation, and the production of the inflammatory cytokines (TNF-α, INF-γ, and IL-6)^76^. TNF-α, a strong proinflammatory cytokine, plays key functions in differentiation, immunity, inflammation, and apoptosis^77^. Besides, Matsumura, et al.^78^ reported that, under inflammatory conditions, hepatocytes become more reactive to TLR-2 ligands leading to TLR-2 up-regulation via LPS, TNF-α, and IL-1β in an NF-κB-dependent manner. Herein, in both hepatic and renal tissues of the food preservatives-exposed groups, a significant up-regulation TLR-4, TLR-2, NF-κB, and TNF-α genes were strongly correlated with a depleted antioxidants content (CAT, SOD, and GSH). Moreover, a strong correlation was evident between the expressions of TLR-4, TLR-2, NF-κB, and TNF-α genes and hepato-renal dysfunction indicators. The earlier findings implied that long-term exposure to BHA, BA, SB, PS, and CP provoked hepatic and renal inflammatory reactions probably via TLRs/NF-κB pathway. Consistent with our results, Yilmaz and Karabay^79^ found that SB boosted NF-κB-p65 protein levels and NF-κB activation of HCT116 colon cancer cells at its cytotoxic concentrations. The significant increase in TNF-α gene expression was previously reported in splenic tissue by Abd-Elhakim et al.^20^ in response to BA, BHA, PS, SB, and CP exposure. In addition, Iheanyichukwu et al.^28^ observed a significant increase in TNF-α gene expression following tartrazine and erythrosine administration implying serious damage or inflammatory activity in the treated rats' kidneys. Elevated expression of TNF-α could be either in response to ROS induction/oxidative stress^80^ or as a result of activation of the TLR-2 and 4 downstream signaling cascades, rather than as a direct result of ROS/oxidative stress induction. The proposed oxidative stress and inflammatory pathways through which the five food preservatives induced hepatorenal injury were presented in Fig. 6.

## Conclusion

In the current study, 90-day oral dosing of five food preservatives (PS, BHA, BA, SB, and CP) in a rat model displayed a significant increase in hepatic enzyme leakage, serum renal damage products levels, depleted antioxidant enzymes, and various histopathological perturbations in renal and hepatic tissues. The up-regulation of hepatic and renal TLR- 4, TLR-2, NF-κB, and TNF-α genes could be probable underlying mechanisms. Boric acid showed the most detrimental impacts on the liver and kidney, followed by BHA and PS. Overall, the current study's findings emphasize the importance of limiting food preservatives and using only the legal limit in manufacturing. Additional research may be required and beneficial in changing the industry's perception of food preservatives as safe components, resulting in more cautious use. More rigorous public and food safety authority surveillance surveys are required to use these chemicals in food production.

## Data Availability

All data generated or analyzed during this study are included in this published article.

## Abbreviations

ALP: Alkaline phosphatase
ALT: Alanine transaminase
AST: Aspartate transaminase
BA: Boric acid
BHA: Butylated hydroxyanisole
CAT: Catalase
CP: Calcium propionate
GSH: Reduced glutathione
MDA: Malondialdehyde
NF-κB: Nuclear factor kappa-light-chain-enhancer of activated B cells
PS: Potassium sorbate
RT-PCR: Real-time quantitative polymerase chain reaction
SB: Sodium benzoate
SOD: Superoxide dismutase
TLR-2: Toll-like receptors 2
TLR-4: Toll-like receptors 4
TNF-α: Tumor necrosis factor-alpha
